# Factors associated with quality of life of people with Myasthenia Gravis

**DOI:** 10.1371/journal.pone.0206754

**Published:** 2018-11-08

**Authors:** Ansuk Jeong, Ju-Hong Min, Yu Kyoung Kang, Juhyeon Kim, Misong Choi, Jin Myoung Seok, Byung Joon Kim

**Affiliations:** 1 Department of Psychology, University of Utah Asia Campus, Yeonsu-gu, Incheon, South Korea; 2 Department of Neurology, Samsung Medical Center, Sungkyunkwan University College of Medicine, Kangnam-gu, Seoul, South Korea; 3 Department of Neurology, Yeouido St. Mary’s Hospital, Yeongdeungpo-gu, Seoul, South Korea; 4 Department of Neurology, Soonchunhyang University Cheonan Hospital, Dongnam-gu, Cheonan, South Korea; University of Birmingham, UNITED KINGDOM

## Abstract

**Purpose:**

As most of patients with Myasthenia Gravis have limitations in their physical functioning, many experience changes in psychological states and often have depression. The objective of the current study was to examine the roles of communication with medical professionals, patients’ loneliness, and patients’ depression, in relation to their effects on the patients’ quality of life.

**Methods:**

For 120 patients with MG of 18 years and older, demographic variables, along with communication with medical professionals, loneliness, depression, and quality of life were measured.

**Results:**

As a result, people suffering from MG experienced lower quality of life when their career has changed due to the illness. At the same time, depression was a significant predictor of their quality of life, both in physical and mental domains.

**Conclusions:**

The implications for clinical settings and the suggestions for future research are discussed.

## Introduction

Myasthenia Gravis (MG) is an autoimmune neuromuscular junction disorder, characterized by fluctuating motor weakness such as ptosis, diplopia, dysarthria, dysphagia, dyspnea, weakness of arms and legs, and fatigue. These symptoms influence daily life of the patients. Ptosis, which is visible, makes the affected self-conscious and thus brings about social withdrawal [1], and diplopia, double vision, is a constant threat to the well-being of the affected while they are awake. Fatigue and other motor weakness hinder the affected from completing basic daily tasks, including taking care of their hygiene [2].

In many cases with chronic illness, depression has been reported to be prevalent, even longitudinally [3]. Considering the interferences of daily functioning due to the symptoms of MG, heightened depression and lowered quality of life (QOL) are not unanticipated [4–12]. Disease’s severity [4], lower education [5], older age [5–6], being woman [7], as well as lower social support [5] were positively correlated with patient’s physical health [4–7], while being optimistic, positive thinking, and sense of humor functioned as protective factors [8] for a better QOL.

Living with a medical condition is stressful and requires effortful adaptation. In a concept analysis of “living with chronic illness,” Ambrosio and colleagues noted that adults with chronic illness go through such psychological status as acceptance, coping, self-management, integration, and adjustment [13]. In another qualitative study with Taiwanese people, it was also noted that living with MG accompanies adjusting to the new norm of living with the chronic condition [14].

In going through those stages, people with chronic illness benefit from the support of family and friends, as was evidenced in research on general health outcomes [15], cancer [16], as well as chronic illness, including MG [5, 14, 17]. Also support of medical professionals is reported to have positive influence on cancer patients [18–19].

In those regards, the current study aims to investigate the relations among the above-mentioned socio-demographic conditions, depression, social support, and QOL in patients with MG. More specifically, the current study employs communication with medical professionals and patients’ loneliness as proxies of the social support and those variables will be examined in relation to their effects on patients’ depression and QOL. Despite its relevance to people with certain chronic condition, including cancer, the patients’ perception of medical professionals’ support has not been studied with regards to its influence on MG patients’ well-being. Regarding the social support, loneliness can be a good index because the visible symptoms of MG can lead the patients to social withdrawal. Combined with the Korean *face*-*saving* culture, which hinders people from discussing their weakness, including medical issues, people with chronic illness may as well experience higher levels of loneliness.

## Material and methods

### Procedures

As soon as the Institutional Review Board of Samsung Medical Center approved the study, researchers on site advertised the participation opportunities in the outpatient clinic and collected the data prospectively. Researchers are medical doctors who are specialized in the field of neuromuscular disorders. When each participant provided his/her signed informed consent, after being explained of the research purpose and procedures by a trained research fellow on site, the participant started completing the survey. If the participant needed an aide, due to their MG symptoms for example, the research assistant on site read out the questions for the participant. Each survey took about 15–25 minutes to complete. As a compensation, a $5 coupon for a coffee house was given to the participants after they completed the survey. The data collection took 6 months from November 2016 to April 2017.

### Participants

Adults (18 years old or above) who are diagnosed with MG and being treated in the outpatient clinic were targeted for recruitment. The diagnosis of MG was made based on the typical history and signs of fluctuating motor weakness, positive acetylcholine-receptor antibody, abnormal decremental responses on a repetitive nerve stimulation test, and definite improvement to acetylcholinesterase inhibitors [20–21]. People with physical and/or mental barriers to complete a questionnaire in Korean language were excluded.

### Measurements

#### Demographic variables

Participants’ age, gender, MG type (ocular or generalized), duration of MG, MG composite score (MGCS) [22–23], Korean MG-activity of daily living (KMG-ADL) [24–25], education, marital status, living arrangement, income, and career change due to MG were measured. MGCS score ranges from 0 to 50, with higher score suggesting more severe symptoms of MG. KMG-ADL score ranges from 0 to 24, with higher score suggesting more interference in daily living activities due to MG, as is validated in Korea [25]. Also career change was coded as binary (“no” as 0 and “retirement,” “leave of absence,” and “moving to different occupation” as 1).

#### Communication with medical professionals

The Chinese Medical Interview Satisfaction Scale [26] was employed to measure the participants’ perception of their communication with the medical professionals. The Medical Interview Satisfaction Scale (MISS) was originally developed for British population [27], and Lam and colleagues revised it (MISS-R) and translated it to Chinese language (C-MISS-R) and validated it among Chinese population. The English version of the C-MISS-R was translated into Korean for the current study. By asking if they “felt understood by my doctor” and “doctor told me all I wanted to know about my illness,” the scale measures how supported the patients feel cognitively and affectively by their doctors. Composed of 8 items, the scale showed moderately high internal consistency in the current study (Cronbach’s *α* = .780). The total score ranges from 8 to 40, with higher score indicating better communication with the professionals.

#### Loneliness

UCLA Loneliness Scale [28] was used to measure how un-supported the patients feel generally. Items include “I have nobody to talk to” and “I feel completely alone.” Composed of 20 items, the scale showed high internal consistency in the current study (Cronbach’s *α* = .881). The total score ranges from 20 to 80, with high score suggesting higher level of loneliness.

#### Depression

The Center for Epidemiological Studies–Depression, developed by Radloff, was used to measure the participants’ depression level. The responses to rate how often they felt during the past week, such as “sad” and “everything I did was an effort,” are summed up. Its 20 items showed high internal consistency in the current study (Cronbach’s *α* = .918). The total score ranges from 20 to 80, with higher score indicating higher level of depression.

#### Quality of life (QOL)

The Short Form Health Survey (SF-36 version 2) was used to measure the participants’ QOL. Responses to 36 questions asking about the health status such as “climbing up the stairs for several floors (was hard due to my illness)” and “I completed less tasks than I wished (due to my illness)” were scored according to the scoring system provided by the developer. As a result, 8 domains were yielded, including Mental Health (MH), Vitality (VT), Social Functioning (SF), General Health (GH), Physical Function (PF), Bodily Pain (BP), Role-Physical (RP), and Role-Emotional (RE), which was categorized into two sets, Physical Composite Summary (PCS) and Mental Composite Summary (MCS). The total scores of PCS and MCS range from 0 to 100, with higher score suggesting higher QOL and less disability.

### Analysis

Hierarchical Multiple Regressions were run with PCS (Physical Composite Summary) and MCS (Mental Composite Summary) of SF-36 as outcome variables for separate analyses respectively. Guided by the current literature on the QOL of people with MG, patient’s socio-demographic variables, such as age, gender, duration of MG, career change, MGCS, and KMG-ADL were entered in the first step [3–7], followed by the patient’s depression, loneliness, and communication with medical professionals in the second step [5, 14, 17–19]. *R*^*2*^ for the whole model and *R*^*2*^ change at each step will be reported. Also, Variance Inflation Factor (VIF) will be used to address the potential collinearity issue.

## Results

A total of 120 patients participated: 48 (40%) were male and 72 (60%) were female. The characteristics of the participants are shown in S1 Table, along with the descriptive statistic results of the variables of interest in the current study.

For Physical Composite Summary, no multicollinearity amongst the variables included in the analyses was found (*VIF* ranging from 1.001 to 1.905). The Hierarchical Multiple Regression yielded two models: Model I with age and career change being significant predictors; and Model II with age, depression, and communication with medical professionals being significant predictors. Considering the *R*^*2*^ of each model, Model II is selected. The specifics are shown in S2 Table.

For Mental Composite Summary, no multicollinearity amongst the variables included in the analyses was found (*VIF* ranging from 1.001 to 1.821). The Hierarchical Multiple Regression yielded two models: Model I with career change and KMG-ADL being significant predictors; and Model II with depression being a significant predictor. Considering the *R*^*2*^ of each model, Model II is selected. The specifics are shown in S3 Table.

## Discussion

### Summary of results

Overall, patients’ demographic variables explained much variance of PCS whereas psycho-social variables added notable amount of explained variance of MCS. Compared with the literature [4–7], gender was not a significant factor in explaining QOL of people with MG in the current study, while age was a significant factor for the patient’s physical QOL: older persons with MG experienced lower physical QOL, which is not surprising.

The socio-demographic factor (Model I) that explained both physical and mental QOL for people with MG in the current study was career change: the participants seemed to be experiencing higher QOL when their career has not changed due to their illness. As the current study employed concurrent measurements, however, the direction between the cause and effect is not clear: it is possible to have their career changed due to their declined physical functioning and QOL; but it is also equally possible to experience lower mental QOL due to the change of career after the diagnosis of MG.

Among the psycho-social factors (Model II) included in the analyses, depression predicted both aspects of QOL negatively: the more depressed the patients were, the lower physical and mental QOL the patients reported. At the same time, communication with medical professionals predicted the physical QOL negatively, with those perceiving less support from medical professionals experiencing higher physical QOL. It is possible that people who are relatively functioning well do not communicate with their medical professionals in-depth.

Loneliness, the variable chosen as a proxy of the lack of social support in the current study, was not found significant in explaining the patient’s QOL. This is contrary to the literature supporting the impact of social support on the QOL of people with chronic condition [5, 14–17]. The rationale behind the choice of loneliness as a proxy of social support, or the lack thereof, was that people eventually would feel “left alone” if they perceive they have “no one to talk to.” In the current study, however, results did not support the rationale. This discrepancy implies the possibility that the perceived lack of social support is not necessarily experienced as loneliness. The potentially divergent nature of the two constructs is worth investigating in the future.

### Implications

The findings of the current study lead to some important implications. First of all, the career change and depression were the most significant predictors of MG patients’ QOL. Physical difficulty due to the illness might have caused the patients to quit working, to take a leave of absence, to change the occupation to what is more manageable, or to retire. This was held true even while the participants’ physical symptoms of MG were moderate in the current study. In other words, the career change due to MG might not necessarily derive from the physical inconvenience. Once diagnosed with a medical condition that does not have a quick and easy solution, people could become more vulnerable to multiple types of manifestation of lower mental QOL, including depression, low self-esteem, and social withdrawal. This vicious cycle of physical functioning, work arrangement, and mental health represents the dilemma of sick role and self-concept. When you are very sick, you get certain support, at the cost of self-esteem. Particularly in a collectivistic culture, including South Korea, however, where *saving face* is a very important part of lifestyle [29], seeking for support or attaining support can be considered as a manifestation of weakness, which is not necessarily considered to be desirable. Therefore, the systemic support for people with chronic illness can be effective when implemented in the workplace considering their experience of the dilemma.

More specifically, assuming career change as one aspect of lowered quality of life, the patients’ efforts to improve their self-esteem and obtain satisfaction even after their career change are needed. For those who go through inevitable career changes, providing programs that include career construction counseling with connections to suitable alternative workplaces can be effective. In addition, participating in psychotherapy, such as having group therapy with people who are facing similar situation, may encourage patients to cope with their mental stress and strengthen their self-esteem.

At the same time, depression was a significant factor for both aspects of the QOL, which needs to be managed. In the field of psycho-oncology, for example, psychosocial intervention programs are run for cancer patients in many different countries and cultures. Even though there is no strong evidence that psychosocial interventions improve QOL and well-being in adults with neuromuscular disorders, including MG, yet, it is also attributed to the paucity of high quality research in this field [30]. To promote the patients’ psychosocial well-being, while their physical condition is managed by pharmaceuticals, certain programs to encourage people to integrate the conditions and to develop meaning out of the experience might be helpful.

### Limitations and suggestions for future research

Even though the current study consisted of a relatively large sample with one diagnosis, the sample was not unbiased. One hundred patients (84% of the participants) finished at least high-school education and the duration of MG was relatively short (*M* = 8.43 months). People with longer years of education tend to actively seek information on the illness and to communicate with their medical professionals rather proactively. The participants’ rate of their perceived support from the medical professionals was actually high (the lowest was 23 and the highest was 40 where the possible range of scores was 8 to 40; *M* = 33.91, *SD* = 3.85), and thus it is possible for the score range to be not variant enough to show the statistical effects in the current study.

The same problem applies to the duration of the illness and age. The participants were experiencing MG for 8.43 months on average (the lowest was 0 and the highest was 33 months), and thus they may be not yet acquainted to MG daily fluctuations and chronic illness state. And their MGCS and KMG-ADL were not high. Considering the participants’ average age, it is reasonable to figure that many of the participants were late onset MG patients. This group of patients who are not yet acquainted to MG fluctuations are biased and thus it would be hard to generalize the findings to a broader population with MG.

Even though the range of MGCS and KMG-ADL in the current study was not large, it was counter-intuitive to see their having no effects on the QOL of the patients when the psycho-social variables were added (Model II), considering that “difficulty chewing solid food” and “shortness of breath at rest” were the two major determinants of poor perceived health [31]. Furthermore, such autoantibodies as anti-muscle receptor tyrosine kinase or anti-titin/RyR antibodies, which suggest different immunological mechanism, and the presence of fatigue, which is reported to be associated with disease severity, ADL, QOL, and depressive state, were not measured in the current study. In those regards, it will be critical in future research to recruit the participants with variable disease duration, to measure different autoantibodies, and to investigate additional factors that are known to influence ADL and QOL, including fatigue [32].

The current study employed concurrent measures, which makes it impossible to infer any cause and effect relationships. To gain a better understanding of the developmental trajectories of the effects of MG, a longitudinal research is suggested. By measuring the same variables over longer period of time at several different time-points, we will be able to investigate the impacts of the illness, both short-term and long-term, so that the psychosocial intervention programs can be implemented at proper points of time from the onset.

Lastly, but not leastly, it should be noted that the current study was conducted in South Korea, whose sample is composed solely of Korean patients. The specificity of the population might have affected the different results from the literature. In the future, therefore, cross-cultural studies are suggested to provide more insight on the effects of diverse socio-cultural contexts on the experience of people with chronic illness.

## Supporting information

S1 Table Characteristics of the participants. (DOCX)

S2 Table Predictors of patient’s PCS (Physical Composite Summary) of the quality of life. (DOCX)

S3 Table Predictors of patient’s MCS (Mental Composite Summary) of the quality of life. (DOCX)
