# Supplementary material for: Factors associated with quality of life of people with Myasthenia Gravis
Source: PLoS One. 2018 Nov 8;13(11):e0206754. doi: 10.1371/journal.pone.0206754 (PMC6226107; doi:10.1371/journal.pone.0206754)
Supplement: S1 Table — (DOCX) [file pone.0206754.s001.docx]

S1 Table. Characteristics of the Participants

| Items | | *n* | Percent |
| --- | --- | --- | --- |
| Gender | Male | 48 | 40 |
|  | Female | 72 | 60 |
| Age | *M (SD)* | 55.65 (15.90) | |
| Income (USD/month) | *M (SD)* | 4000 (3780) | |
| Living arrangement | Alone | 27 | 22.5 |
|  | With spouse | 82 | 68.3 |
|  | With spouse and children | 51 | 42.9 |
|  | With spouse and parents | 18 | 15.0 |
|  | With spouse, children, and parents | 3 | 2.5 |
| Education | No schooling | 1 | 0.8 |
|  | Primary school | 9 | 7.6 |
|  | Middle school | 9 | 7.6 |
|  | High school | 46 | 38.7 |
|  | College/University | 46 | 38.7 |
|  | Post-graduate | 8 | 6.7 |
| Marital Status | Single | 19 | 16.2 |
|  | Married | 86 | 73.5 |
|  | Divorced | 4 | 3.4 |
|  | Widowed | 7 | 6.0 |
| Career Change  due to illness | No change | 75 | 62.5 |
|  | Leave of absence | 16 | 13.3 |
|  | Change to different occupation | 4 | 3.3 |
|  | Retirement | 9 | 7.5 |
| MG* Type | Ocular | 52 | 43.3 |
|  | Generalized | 68 | 56.7 |
| Duration of MG (months) | *M (SD)* | 8.43 (7.41) | |
| MGCS* | *M (SD)* | 3.74 (3.86) | |
| KMG-ADL* | *M (SD)* | 3.18 (2.63) | |
| CMISS-R* | *M (SD)* | 33.91 (3.85) | |
| Loneliness | *M (SD)* | 36.28 (10.40) | |
| Depression | *M (SD)* | 34.12 (11.07) | |
| PCS* | *M (SD)* | 46.13 (8.08) | |
| MCS* | *M (SD)* | 46.85 (11.02) | |

* M, mean; SD, standard deviation; USD, U.S. dollars; MG, myasthenia gravis; MGCS, myasthenia gravis composite score; KMG-ADL, Korean myasthenia gravis- activity of daily living; CMISS-R, Chinese Medical Interview Satisfaction Scale Revised; PCS, Physical Composite Summary; MCS, Mental Composite Summary.
