# Supplementary material for: Factors associated with quality of life of people with Myasthenia Gravis
Source: PLoS One. 2018 Nov 8;13(11):e0206754. doi: 10.1371/journal.pone.0206754 (PMC6226107; doi:10.1371/journal.pone.0206754)
Supplement: S2 Table — (DOCX) [file pone.0206754.s002.docx]

S2 Table. Predictors of Patient’s PCS (Physical Component Summary) of the Quality of Life

| Predictors | Model I | |  | Model II | |
| --- | --- | --- | --- | --- | --- |
|  | *β* | *p* |  | *β* | *p* |
| Age | -.292 | .007 |  | -.351 | .001 |
| Gender | -.163 | .162 |  | -.125 | .265 |
| Duration of MG* | .034 | .752 |  | .037 | .715 |
| Career change | .287 | .014 |  | .177 | .112 |
| MGCS* | .024 | .915 |  | .008 | .970 |
| KMG-ADL* | -.119 | .601 |  | -.015 | .945 |
| Depression |  |  |  | -.435 | .004 |
| Loneliness |  |  |  | .034 | .805 |
| Communication with medical professionals |  |  |  | -.204 | .048 |
| *R^2^* change |  | |  | .143 | |
| *R^2^* | .263 | |  | .406 | |

* MG, myasthenia gravis; MGCS, myasthenia gravis composite score; KMG-ADL, Korean myasthenia gravis- activity of daily living;
