# Supplementary material for: Factors associated with quality of life of people with Myasthenia Gravis
Source: PLoS One. 2018 Nov 8;13(11):e0206754. doi: 10.1371/journal.pone.0206754 (PMC6226107; doi:10.1371/journal.pone.0206754)
Supplement: S3 Table — (DOCX) [file pone.0206754.s003.docx]

S3 Table. Predictors of Patient’s MCS (Mental Component Summary) of the Quality of Life

| Predictors | Model I | |  | Model II | |
| --- | --- | --- | --- | --- | --- |
|  | *β* | *p* |  | *β* | *p* |
| Age | .043 | .683 |  | -.041 | .585 |
| Gender | .056 | .629 |  | .046 | .583 |
| Duration of MG* | .130 | .223 |  | .117 | .126 |
| Career change | .238 | .040 |  | .023 | .782 |
| MGCS* | .151 | .505 |  | .125 | .427 |
| KMG-ADL* | -.528 | .022 |  | -.309 | .058 |
| Depression |  |  |  | -.754 | .000 |
| Loneliness |  |  |  | .064 | .541 |
| Communication with medical professionals |  |  |  | -.022 | .774 |
| *R^2^* change |  | |  | .395 | |
| *R^2^* | .269 | |  | .664 | |

* MG, myasthenia gravis; MGCS, myasthenia gravis composite score; KMG-ADL, Korean myasthenia gravis- activity of daily living
